# Screening for Subclinical Atherosclerosis in Patients with Familial Hypercholesterolemia: Insights and Implications

**DOI:** 10.3390/jcm14020656

**Published:** 2025-01-20

**Authors:** Muhammed Furkan Deniz, Baris Guven, Abdullah Omer Ebeoglu, Omer Burak Gul, Ali Nayir, Pelinsu Ozkan, Zubeyir Bulat, Ibrahim Turk, Ozlem Demirelce, Husamettin Alper Kimyonok, Habibe Deniz, Murat Kazım Ersanli, Veysel Oktay, Dildar Konukoglu, Umit Yasar Sinan

**Affiliations:** 1 Institute of Cardiology, Istanbul University-Cerrahpaşa, 34098 Istanbul, Türkiye; drfurkandeniz@gmail.com (M.F.D.); guvenbariss@gmail.com (B.G.); ebeoglu995@gmail.com (A.O.E.); omerburakgul06@gmail.com (O.B.G.); ali.nayir@hotmail.com (A.N.); hunkarpelinsu@gmail.com (P.O.); zbyrbulat@gmail.com (Z.B.); mersanli@gmail.com (M.K.E.); drvoktay@gmail.com (V.O.); dkonuk@yahoo.com (D.K.); 2 Istanbul Training and Research Hospital, 34098 Istanbul, Türkiye; ibrahimturk.md@gmail.com (I.T.); ozlem24@msn.com (O.D.); alperkimyonok@yahoo.com (H.A.K.); 3 Bahçelievler State Hospital, 34186 Istanbul, Türkiye; habibeozdemirdeniz@gmail.com

**Keywords:** familial hypercholesterolemia, subclinical atherosclerosis, medication adherence

## Abstract

**Background/Objectives**: Familial hypercholesterolemia (FH) is a monogenic dyslipidemia that leads to early cardiovascular events. Subclinical atherosclerosis refers to the formation of atheromatous plaques in arterial beds before any clinical events. In our study, we investigated the presence, extent, and independent predictors of subclinical atherosclerosis among patients diagnosed with FH. **Methods**: This was a single-center, prospective, and cross-sectional study. This original study included 215 patients diagnosed with FH from a cohort of 1145 individuals assessed according to the Dutch Lipid Clinical Network (DLCN) criteria. Carotid and femoral ultrasonography were performed, and the coronary artery calcium score was measured to screen for subclinical atherosclerosis. Apolipoprotein A-I, apolipoprotein B, and lipoprotein (a) were analyzed using the nephelometric method. **Results**: The study cohort comprised 136 females (63%) with a mean age of 54 (43–62) years. The stigmata rate was 18%. The rate of statin use during subclinical atherosclerosis screening was 32% and only eight patients (4%) attained LDL-C values < 70 mg/dL. Subclinical atherosclerosis was observed in 148 patients (69%), with rates of 48%, 47.5%, and 40.5% in the coronary arteries, carotid bifurcation, and femoral bifurcation, respectively. Advanced age, male sex, high pretreatment low-density lipoprotein-cholesterol (LDL-C) level, diabetes, and a low Apo A-I/Apo B ratio were identified as independent predictors of subclinical atherosclerosis. Lp(a) levels ≥ 30 mg/dL predicted coronary atherosclerosis, while diabetes and low Apo A-I/Apo B ratios predicted carotid atherosclerosis, and smoking predicted femoral atherosclerosis. **Conclusions**: Subclinical atherosclerosis is prevalent, and medication adherence remains suboptimal among FH patients. Screening for subclinical atherosclerosis may impact the treatment strategies, via an increase in physician commitment to treatment protocols and improving patient compliance.

## 1. Introduction

Familial hypercholesterolemia (FH) is an autosomal dominant disease that leads to early cardiovascular events. It manifests in two forms, heterozygous and homozygous. Diagnosis is usually confirmed by identifying pathogenic mutations. However, a causative genetic variant can only be identified in 60–80% of patients with FH, thereby limiting its diagnostic utility in patients without identifiable mutations. Furthermore, due to financial limitations, genetic testing cannot be performed for all patients in developing countries like Türkiye. Therefore, validated and clinically proven scoring systems are particularly valuable in these countries, where the widespread implementation of genetic testing is limited by financial constraints. The Dutch Lipid Clinical Network (DLCN) score is currently the most widely used scoring system for diagnosing FH. Despite the elevated cardiovascular risk associated with FH, the level of awareness regarding the disease remains significantly low and a substantial proportion of patients fail to achieve the recommended treatment goals. This suboptimal management is an important factor contributing to the high incidence of major adverse cardiovascular events (MACEs) observed in this population. Hence, prompt diagnosis, early detection of atherosclerosis, and initiation of lipid-lowering therapy are pivotal for saving the lives of patients with FH [1,2,3,4].

Subclinical atherosclerosis refers to the formation of atheromatous plaques in arterial beds, such as the coronary, carotid, or iliofemoral arterial segments, before symptomatic events related to the atherosclerotic process. Subclinical atherosclerosis is an early indicator of atherosclerotic burden and can be identified using non-invasive imaging techniques. The progression and morphological characteristics of atherosclerotic plaques are dynamically influenced by temporal progression and advancing age, exhibiting region-specific pathophysiological behaviors within distinct arterial beds. Plaques may stabilize in certain vascular beds while becoming increasingly vulnerable in others. This variability emphasizes the critical importance of conducting comprehensive assessments of atherosclerosis across multiple vascular beds to understand disease progression and associated risks. Moreover, the involvement of multiple vascular beds provides insight into the extent of subclinical atherosclerosis, and assessing various beds collectively enables the accurate measurement of the overall atherosclerotic burden [5,6,7,8,9].

The development of effective strategies to improve medication adherence and enhance awareness of FH is critically important. There are only a few studies in the literature on subclinical atherosclerosis and familial hypercholesterolemia involving small populations; however, no prior studies have been conducted in Türkiye. In our study, we investigated the presence, extent, and independent predictors of subclinical atherosclerosis among patients diagnosed with FH based on the DLCN score.

## 2. Materials and Methods

**Figure 1:**
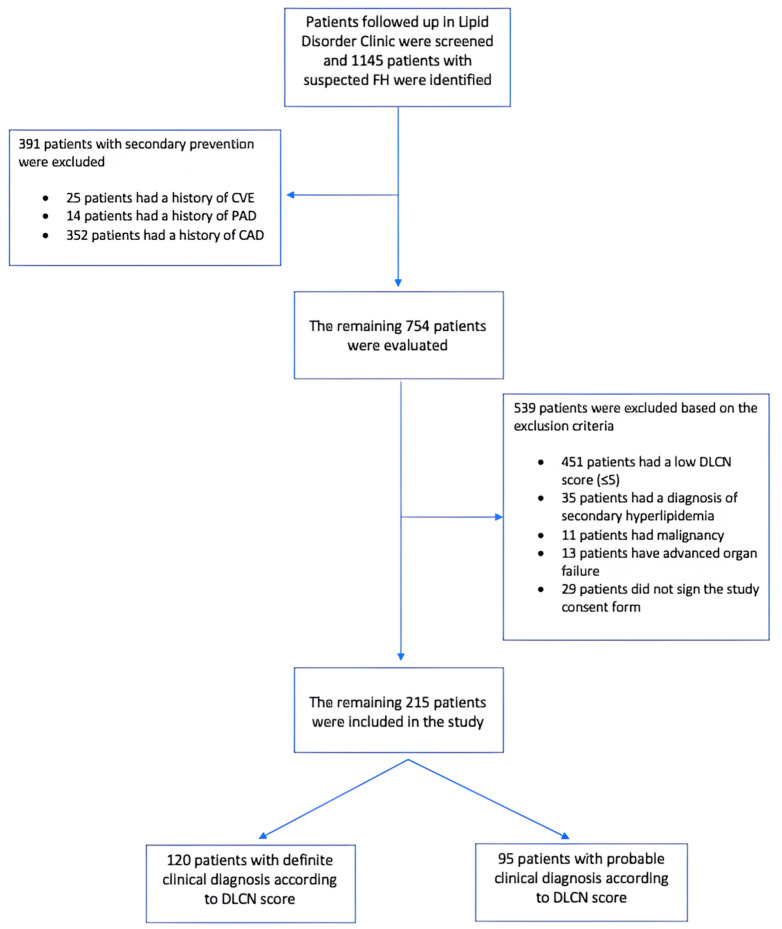
Flow chart of this study.

This was a single-center, prospective, cross-sectional study. Patients managed at the outpatient clinic of our hospital underwent screening based on the DLCN criteria. Among these patients, those aged 18 years or older who were clinically diagnosed with FH according to the DLCN criteria were included in this study. Patients were stratified into two groups. Individuals with a DLCN score greater than 8 were identified as definite FH, whereas those with scores ranging from 6 to 8 were categorized as probable FH. The exclusion criteria were individuals under 18 years of age, patients with secondary diagnoses potentially leading to hypercholesterolemia (such as hypothyroidism, nephrotic syndrome, pregnancy, Cushing’s syndrome and those receiving corticosteroids or immunosuppressive therapy), individuals with a history of secondary prevention (history of atherosclerotic cardiovascular disease [ASCVD]), and patients with advanced organ failure or malignancy. The term ASCVD is defined as a history of myocardial infarction, coronary angioplasty, or coronary artery bypass grafting (CABG); detection of carotid artery disease on imaging following a cerebrovascular event; or a history of peripheral artery disease. Demographic information and clinical characteristics of the patients were recorded. Laboratory parameters were analyzed using blood samples collected within the preceding month, followed by a fasting period of at least 12 h. The pre- and post-treatment lipid levels of patients receiving lipid-lowering therapy were recorded separately. A flowchart of this study is shown in Figure 1.

### 2.1. Carotid Ultrasonography (USG)

Ultrasonographic examination of the carotid arteries was conducted using a Samsung RS85 Prestige system (Samsung Electronics Health & Medical Equipment and Samsung Medison, Seul, Republic of Korea), along with a linear probe (model LA2-14A). The patients were positioned supine with their heads rotated at a 45-degree angle towards the contralateral side. The left and right common carotid arteries (within the first 10 mm before the bifurcation), the carotid bifurcation region (bulbus carotid), and the proximal segment (first 10 mm) of the internal and external carotid arteries were imaged. All anatomical regions were meticulously examined from diverse viewing angles, encompassing both cross-sectional and longitudinal perspectives. Measurements were conducted independently by two expert radiologists who were blinded to the patients’ clinical characteristics. Carotid plaques were assessed according to the criteria outlined in the European Mannheim Consensus [10]. Focal thickening characterized by a 0.5 mm indentation into the lumen, thickening representing 50% of the surrounding intima-medial thickness (IMT), and thickening resulting in an IMT of >1.5 mm were identified as carotid plaques. The maximum plaque thickness and plaque structure were also evaluated using carotid USG. To determine the maximum plaque thickness, all plaque lesions visualized in the right and left carotid arteries were evaluated, and the thickest part of the lesion was recorded as the maximum plaque thickness. Plaque characteristics were classified according to the Gray–Weale classification, which is based on the echogenicity of the adjacent tissues. The plaques were categorized into the following four subgroups: type 1 (totally hypoechoic), type 2 (predominantly hypoechoic), type 3 (predominantly hyperechoic), and type 4 (totally hyperechoic). The number of patients with type 2 and type 3 plaques was limited; therefore, these groups were combined into the mixed plaques group [11].

### 2.2. Femoral Ultrasonography

The femoral arteries were examined ultrasonographically using a Samsung RS85 Prestige system (Samsung Electronics Health & Medical Equipment and Samsung Medison, Seul, Republic of Korea) with a linear probe (model LA2-14A). Plaques were investigated within the 20 mm segment of the bifurcation region located at the confluence of the common femoral artery with the superficial and deep femoral arteries. All anatomical regions were comprehensively examined from both cross-sectional and longitudinal perspectives. The measurement methodologies for plaque assessment via femoral ultrasonography were adapted from the established criteria for carotid plaque measurement. Two expert radiologists blinded to the clinical profiles of the patients performed all measurements. The plaque presence, characterization, and maximum thickness measurements were conducted in a manner consistent with the carotid plaque assessment protocols.

### 2.3. Coronary Artery Calcium (CAC) Score

CAC scores were measured using a 128-slice computed tomography scanner (Philips Ingenuity 128 Circular Edition; Philips Medical Systems, Best, Amsterdam, The Netherlands). Contrast material was not administered during the examination. The imaging protocol involved a slice thickness of 3 mm, scenogram parameters set at 80–200 mAs and 120 kV, and a gantry rotation time of 0.3 s. After acquiring a topogram image with the patient in the supine position, single breath-hold imaging lasting approximately 8–10 s was performed, covering the entire heart from 1 cm below the carina. Cardiac CT images were simultaneously reviewed by two expert radiologists blinded to the clinical characteristics of the patients. The Philips Intellispace Portal Version 11 Workstation software was used for the review. CAC measurements were conducted according to the Agatston scoring criteria. Lesions demonstrating a CT density > 130 Hounsfield Units (HU) within an area > 1 mm^2^ and spanning 2–3 adjacent pixels were classified as calcification. The area, density, and calcium score of each lesion identified on axial sections were automatically computed using dedicated software. The total CAC was determined by summing the individual calcium scores of the left main coronary artery (LMCA), left anterior descending coronary artery (LAD), circumflex artery (Cx), and right coronary artery (RCA). Coronary atherosclerosis was defined as a CAC score of ≥1.

### 2.4. Measurement of Laboratory Parameters

To measure the levels of Apo A-I, Apo B, and lipoprotein (Lp)(a), blood samples were obtained from patients after a 12 h fasting period. The samples were then centrifuged at 4000 rpm for 15 min. After centrifugation, the separated plasma and sera were frozen at −80 °C and stored for later analysis. A nephelometric method was used to measure the levels of Apo A-I, Apo B, and Lp(a). Serum samples, which had been previously frozen at −80 °C, were thawed before analysis. Following the completion of appropriate controls and calibrations of the Siemens BNProspec^®^ (Siemens Healthineers, Erlangen, Germany) analyzer, the thawed sera were analyzed.

This study received ethical approval from the Istanbul University-Cerrahpasa Ethics Committee, with approval granted under decision dated 19 September 2023, bearing reference number E-83045809-604.01.01-789808. The research adhered to the ethical principles outlined in the Declaration of Helsinki, and informed consent was obtained from all participants.

### 2.5. Statistical Analysis

The Statistical Package for Social Sciences (SPSS) version 23 (SPSS Inc., Chicago, IL, USA) was used for statistical analyses. The Kolmogorov–Smirnov and Shapiro–Wilk tests were performed to determine the distribution patterns of numerical variables. Normally distributed variables were analyzed using the Student’s *t*-test or one-way analysis of variance (ANOVA) and are presented as means and standard deviations. Variables that did not show a normal distribution were analyzed using the Mann–Whitney U or Kruskal–Wallis test and expressed as median and interquartile range (between the 25th and 75th percentiles). Categorical variables were analyzed using the chi-square or Fisher’s exact test and presented as frequency and percentage distributions. The relationships among subclinical atherosclerosis, coronary atherosclerosis, carotid, and femoral atherosclerosis, and other variables were examined using multivariable logistic regression. The Hosmer–Lemeshow test was used to assess the model’s goodness of fit. The association between the extent of subclinical atherosclerosis and numerical variables was analyzed using the Pearson and Spearman correlation tests. The receiver operating characteristic (ROC) curve analysis was conducted to assess the predictive value of significant numerical variables identified in the correlation analysis for the dependent variable and to determine the corresponding cut-off levels. The significance threshold was set at *p* < 0.05 for all analyses.

## 3. Results

After screening, 1145 patients with suspected FH were identified. The patients were analyzed to determine their eligibility based on this study’s inclusion and exclusion criteria. Finally, 215 patients with no history of ASCVD were included in this study (Figure 1). Among them, 120 and 95 patients had definite and probable FH, respectively, according to the DLCN scoring. The study cohort comprised 136 females (63%) and 79 males (37%), with a mean age of 54 (43–62) years. The most prevalent comorbidities were hypertension (41%) and diabetes (13.5%). Physical examinations revealed stigmata in 38 patients (18%). Genetic test results were available for 46 patients (42 patients had LDL-R gene mutations and 4 patients had Apo B gene mutations). Among the study population, 17% reported using aspirin, 32% reported using statins, and 19.5% reported using potent statins (10%, rosuvastatin; 9.5%, atorvastatin). Additionally, 3.5% reported using ezetimibe, and 1% reported using proprotein convertase subtilisin/kexin type 9 (PCSK-9) inhibitors. The clinical characteristics of the study population and respective study groups are presented in Table 1.

When evaluating the mean pretreatment lipid levels of patients, we observed that low-density lipoprotein-cholesterol (LDL-C) was 252 mg/dL, Total cholesterol (TC) was 329 mg/dL, triglyceride (TG) was 190 mg/dL, high-density lipoprotein-cholesterol (HDL-C) was 55 mg/dL, and non-HDL-C was 273 mg/dL. The rate of statin use during the subclinical atherosclerosis screening was 32%, with only 45 patients (21%) achieving a 50% reduction in LDL-C levels. Furthermore, only eight patients (4%) attained LDL-C values below 70 mg/dL. These findings highlight that within this high-risk patient group, both the rate of statin use and the attainment of treatment goals are notably low.

Patients with definite FH were older, on average, and all patients with stigmata were included in this group. The rates of aspirin, statin, potent statin, and ezetimibe use were significantly higher in patients with definite FH. While treatment differences were significant, the achievement of target LDL-C levels did not differ between the groups. Pretreatment lipid levels and levels of certain biomarkers, such as Lp(a) and Apo B, were higher in the definite FH group. Females generally had higher lipid and biomarker levels than males. Other laboratory parameters showed no significant differences between the groups.

Subclinical atherosclerosis was observed in 148 patients (69%), with rates of 48%, 47.5%, and 40.5% in the coronary arteries, carotid bifurcation, and femoral bifurcation, respectively. Atherosclerosis occurred in one, two, or all three regions in 25%, 27%, and 17% of the cases, respectively. Type 4 plaques were most prevalent in the carotid and femoral arteries (62% and 71%, respectively), followed by mixed type (20% and 16%, respectively), and type 1 plaques (18% and 13%, respectively). Over half of the patients had a maximum plaque thickness ≥ 1.5 mm in both arterial beds (53% and 54%). In 188 patients who underwent CAC score assessment, 48% had a score of ≥1, with 11% having a score of ≥100. Significant differences were observed between the groups in terms of femoral and coronary atherosclerosis but not in terms of carotid atherosclerosis (Table 2).

The group with subclinical atherosclerosis was older and had higher pretreatment levels of LDL-C, TC, non-HDL-C, and Apo B. However, Lp(a) and Apo A-I levels and the Apo A-I/Apo B ratio were similar between groups. Patients with subclinical atherosclerosis had higher rates of hypertension and diabetes; however, no significant differences were found in statin use between the two groups.

Variables correlated with the extent of subclinical atherosclerosis were assessed based on the affected vascular regions. While the age, femoral, and carotid plaque thicknesses, and CAC score showed strong correlations, the pretreatment LDL-C and non-HDL-C levels and DLCN scoring had weak correlations (Table 3).

**Figure 2:**
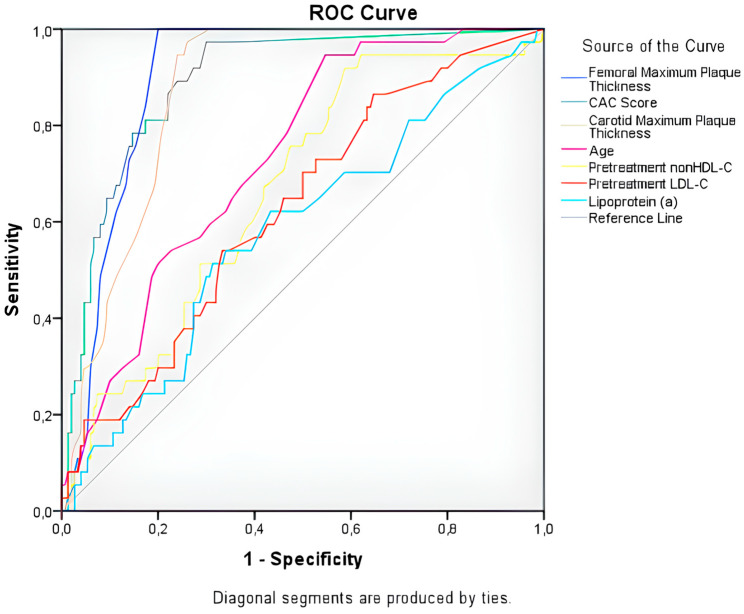
ROC curve analysis.

The study population was stratified into the definite and probable diagnosis groups. Correlation analyses for subclinical atherosclerosis were conducted separately for each group. Both groups showed strong correlations between age, femoral, and carotid plaque thicknesses, and CAC score and the extent of subclinical atherosclerosis. Receiver operating characteristic (ROC) analysis was used to determine the cutoff values, sensitivity, and specificity of these correlated variables. The ROC curve is shown in Figure 2, and the corresponding cutoff values, along with the sensitivity and specificity ratios, are presented in Table 4.

Multiple logistic regression analysis identified several predictors of subclinical atherosclerosis, including advanced age, male sex, high pretreatment LDL-C level, low Apo A-I/Apo B ratio, and diabetes. Interestingly, sex and the Apo A-I/Apo B ratio, which were initially insignificant in the univariable analysis, became significant in the multivariable analysis. Conversely, hypertension and diabetes were initially significant but lost significance. When examining atherosclerosis-prone regions separately, advanced age, male sex, and high pretreatment LDL-C levels were consistent predictors. Additionally, Lp(a) levels > 30 mg/dL predicted coronary atherosclerosis, diabetes, and low Apo A-I/Apo B ratios predicted carotid atherosclerosis, and smoking predicted femoral atherosclerosis (Table 5).

## 4. Discussion

In our study of patients with FH, a high rate of subclinical atherosclerosis (69%) was observed, with similar rates across the coronary (48%), carotid (48%), and femoral (41%) regions. Independent predictors of subclinical atherosclerosis in patients with FH included advanced age, male sex, high pretreatment LDL-C level, presence of diabetes, and low Apo A-I/Apo B ratio. Advanced age, male sex, and high pretreatment LDL-C levels were consistent predictors across all vascular regions. Lp(a) levels ≥ 30 mg/dL predicted coronary atherosclerosis, while diabetes and low Apo A-I/Apo B ratios predicted carotid atherosclerosis, and smoking predicted femoral atherosclerosis. Correlation analysis showed strong associations between advanced age, femoral, and carotid plaque thicknesses, CAC scores, and extent of subclinical atherosclerosis.

In patients with FH, the risk of ASCVD is notably elevated, as demonstrated by studies such as the CASCADE FH Registry and SAFEHEART trials [12,13]. In the CASCADE FH Registry, 3.6% of patients aged > 20 months experienced ASCVD events, and an older age, male sex, low HDL-C level, diabetes, and hypertension were significant factors [12]. In the SAFEHEART study, which followed patients with FH for 5.5 years, ASCVD events occurred in 5.6% of the cases. A predictive model in the SAFEHEART study highlighted an advanced age, male sex, history of ASCVD, high blood pressure, high body mass index (BMI), smoking, high LDL-C levels, and high Lp(a) levels as independent predictors of future major adverse cardiovascular event (MACE) risk [12]. Familial hypercholesterolemia increases the risk of ASCVD, which is compounded by traditional risk factors. Guidelines underscore screening for subclinical atherosclerosis and achieving target LDL-C levels in managing the ASCVD risk in patients [2,3].

Patients with FH are expected to have a high prevalence of subclinical atherosclerosis because of their elevated ASCVD risk. Mattina et al. assessed 154 patients with FH for subclinical atherosclerosis and identified its presence in 83% of patients. The rates of CAC, carotid plaques, and femoral plaques were 62%, 55%, and 56%, respectively, with no significant differences in atherosclerosis in the coronary, carotid, and femoral regions; however, coronary atherosclerosis was higher in males, while carotid atherosclerosis was higher in females [14]. In our study, subclinical atherosclerosis was found in 69% of the patients and was most common in the coronary arteries, similar to results from a previous study. However, contrary to that study, the rates of coronary, femoral, and carotid atherosclerosis were higher in females, possibly because of the higher proportion of females in the definite FH group. Moreover, the mean age of female subjects (58 years) compared to male subjects (44 years) may have contributed to this finding. The advanced age of female subjects, along with the age-related decline in estrogen—a protective factor in females—may partly explain the numerically higher incidence of subclinical atherosclerosis observed in females. Additionally, while 33% of the patients in the previous study had atherosclerosis in all three regions, the rate was 25% in our study. These findings highlight the high prevalence and frequency of subclinical atherosclerosis in patients with FH and provide valuable insights into the occurrence of atherosclerosis in this population.

However, different results have been obtained when examining the regions in which subclinical atherosclerosis develops. In the Aragon Workers’ Health Study (AWHS) by Laclaustra et al., 72% of the participants had subclinical atherosclerosis, and the femoral region was the most common site. Active smoking was the strongest predictor of femoral and carotid plaques, whereas hypertension was associated with CAC, the strongest predictor of coronary atherosclerosis [15]. In our study, hypertension was initially associated with subclinical and coronary atherosclerosis in univariable analysis but lost significance in multivariable analysis. Smoking was an independent predictor of femoral atherosclerosis but not of subclinical atherosclerosis. Interestingly, femoral plaques in the AWHS study showed a higher sensitivity in predicting CAC than carotid plaques. In our analysis, femoral plaques were independent predictors of coronary atherosclerosis, unlike carotid plaques, underscoring their importance in predicting coronary atherosclerosis. It is important to emphasize that in both the previously referenced studies and our study, coronary atherosclerosis was evaluated using CAC scoring. However, as the CAC score is limited to detecting calcified plaques and does not account for non-calcified plaques, it may lead to an underestimation of the true coronary atherosclerotic burden.

In other studies, the predictors of subclinical atherosclerosis varied. Chan et al. found that advanced age, male sex, hypertension, and pretreatment LDL-C and Lp(a) levels were independent predictors of coronary artery disease (CAD) [16]. Allard et al. identified male sex, diabetes, a family history of CVD, and high Lp(a) levels as predictors of cardiovascular events (CVE) [17]. Similarly, in our study, an advanced age, male sex, pretreatment LDL-C levels, and Lp(a) levels ≥ 30 mg/dL were independent predictors of coronary atherosclerosis. These findings emphasize the importance of considering multiple risk factors when assessing the ASCVD risk in patients with FH.

Conflicting results have been reported regarding the relationship between high plasma Lp(a) levels and CAD in patients with FH. Some studies have reported that a high Lp(a) level is an independent risk factor for CAD in patients with FH, while others have reported no relationship between Lp(a) levels and CAD [17,18,19].

Chan et al. divided 390 patients with FH into two groups based on the presence of CAD. The mean Lp(a) level was 42 mg/dL, and the Lp(a) level was significantly higher in patients with a history of CAD [16]. In addition, Alonso et al. investigated the relationship between Lp(a) levels and cardiovascular disease (CVD) in patients with FH and their non-FH relatives. The average Lp(a) level was 24 mg/dL, and 30% of patients had levels > 50 mg/dL. These higher levels were significantly associated with patients with FH who had a history of CVD, compared to those without. Notably, the Lp(a) levels between males and females were not significantly different. Additionally, among patients with FH and a history of CVD, approximately 46% have Lp(a) levels > 50 mg/dL [20]. Nenseter et al. observed no difference in Lp(a) levels between males and females [21]. In the RELACS study conducted by Cesaro et al., 774 patients with acute coronary syndrome (ACS) were included and stratified into three groups based on their Lp(a) levels. The study found that higher Lp(a) levels were associated with a younger age at the first coronary event. Additionally, it was demonstrated that Lp(a) acts as an accelerator of atherosclerotic cardiovascular disease (ASCVD) and is linked to complex coronary artery disease. These findings highlight Lp(a) as a significant risk factor for atherosclerosis and emphasize the need for more aggressive lipid-lowering therapies in primary prevention settings [22]. In our study, the mean Lp(a) level was 16 mg/dL, 32% of patients had Lp(a) levels ≥ 30 mg/dL, and 19.5% had levels ≥ 50 mg/dL. Higher Lp(a) levels were observed in patients with subclinical atherosclerosis, with significantly higher levels in females, likely due to the sex distribution. Multivariable regression analysis identified an Lp(a) level ≥ 30 mg/dL as an independent predictor of coronary atherosclerosis. As demonstrated in the RELACS study, patients with elevated Lp(a) levels require intensified lipid-lowering therapy [21]. Consistently, our study identified a significant association between elevated Lp(a) levels and subclinical coronary atherosclerosis. These findings underscore the critical importance of implementing aggressive lipid-lowering strategies in this patient population to reduce cardiovascular risk.

Advanced age is an important parameter in assessing the risk of ASCVD. The SCAPIS study conducted by Bergström et al. showed that coronary atherosclerosis starts approximately 10 years later in females, and the CAC score is a good indicator of the presence of coronary atherosclerosis [23]. In a study led by Walus-Miarka et al., including 154 patients diagnosed with definite and possible FH according to the Simon–Broome clinical score, patients with detectable carotid plaques were older [24]. In our study, the mean age of individuals with subclinical atherosclerosis was 10 years older than that of other individuals. Therefore, advanced age is an important predictor for the presence of subclinical atherosclerosis.

In our study, females generally had higher lipid profile levels. Moreover, Lp(a) levels, Apo A-I levels, and the Apo A-I/Apo B ratio were significantly higher in female subjects. The protective role of estrogens is well-established and is considered a major factor contributing to the lower incidence of cardiovascular disease in premenopausal individuals. Estrogens have been shown to enhance LDL receptor activity, leading to reductions in total cholesterol and LDL-C levels [14]. The elevated mean age of the female subjects, combined with hormonal influences, may have contributed to the observed differences in lipid parameters.

In both national and international studies, despite the classification of patients with FH as high risk for ASCVD, medication usage rates often fell short of the target values. Ray et al. analyzed 4112 patients receiving lipid-lowering therapy, and approximately 5% were diagnosed with FH. Only 25% of high-risk primary prevention patients and 11% of very high-risk patients achieved the target LDL-C levels [25]. Similarly, Kayikcioglu et al. examined 1071 patients suspected of having FH and found treatment rates at 42% and treatment goal attainment rates of 2.1% [26]. In another study, additional factors contributing to the underutilization of statin therapy were identified, including lack of motivation, side effects, and running out of medication [27]. According to data from the U.S. National CASCADE FH Registry, 92.8% of patients were receiving lipid-lowering therapy at the time of enrollment, but only 73.7% were on statin treatment. The most commonly reported reasons for not using statins were intolerance or allergy (77%) and patient preference (10%). And only 10.6% of patients achieved the treatment goal of an LDL-C concentration below 70 mg/dL [12]. Other studies indicate that the reasons for inadequate drug use are multifactorial and occur at multiple levels, ranging from insufficient prescribing practices at the clinician level to non-adherence at the patient level [28]. In our study, medication usage and achievement of treatment goal rates were notably low, similar to those in these studies. However, the factors associated with medication non-adherence were not investigated in this study. Furthermore, we expect that these rates and patients’ medication adherence may improve after screening for subclinical atherosclerosis. Demonstrating subclinical atherosclerosis in patients with FH may enhance medication adherence and goal attainment.

Our study had several limitations. Firstly, the cross-sectional design and single-center setting may limit its generalizability to broader populations. Secondly, subgroup analyses were not feasible due to the limited sample size in our study. Future large-scale multicenter studies are necessary to enable robust subgroup analyses. Thirdly, only 21% of patients had genetic test results. Not conducting genetic testing for all participants deprived us of valuable insights into the genetic basis of FH and constrained our ability to establish genotype–phenotype correlations. Genetic data not only enhance the validation of clinical diagnoses but also provide crucial insights into patient-specific therapeutic responses and long-term risk stratification. Future research should prioritize expanding access to genetic screening. Furthermore, we did not include a control population for comparison with FH patients. Additionally, with only 32% of the population taking medications, the interpretation of the results may be affected. The absence of contrast-enhanced coronary CT angiography increased the risk of missing noncalcified plaques. Future studies should consider multicenter and prospective designs to address these limitations and provide more detailed and reliable results.

## 5. Conclusions

Subclinical atherosclerosis, as observed in this study, is prevalent among patients with FH. Early diagnosis coupled with the prompt initiation of lipid-lowering therapy is paramount for improving outcomes.

Despite the elevated cardiovascular risk in patients with FH, medication adherence remains suboptimal, with few patients achieving treatment goals. Screening for subclinical atherosclerosis may improve medication adherence, adoption of preventive measures, and it may increase physician commitment to treatment protocols. Thus, detecting subclinical atherosclerosis may be pivotal in optimizing treatment strategies for patients with FH.

## Figures and Tables

**Table 1:** Clinical characteristics and laboratory profiles of the study population.

Variables	Study Population (*n*: 215)	Definite Clinical Diagnosis (DLCN Score > 8) (*n*: 120)	Probable Clinical DiagProbable Clinical Diagnosis (DLCN Score: 6–8) (*n*: 95)	*p* Value
Age (years)	54 (43–62)	55.5 (47.3–62)	51 (41–62)	**0.028**
Gender n (%), (female)	136 (63)	81 (67.5)	55 (58)	0.147
BMI (kg/m^2^)	27.5 (24.8–30.5)	27.6 (25–30.5)	27.3 (25–30.4)	0.595
Obesity *, *n* (%)	57 (26.5)	32 (27)	25 (26.5)	0.954
Family CAD History, *n* (%)	146 (68)	103 (86)	43 (45)	**<0** **.** **001**
Smoking, *n* (%)	113 (53)	66 (55)	47 (49.5)	0.420
Stigmata, *n* (%)	38 (18)	38 (32)	0 (0)	**<0** **.** **001**
Hypertension, *n* (%)	89 (41.5)	55 (46)	34 (36)	0.138
Diabetes, *n* (%)	29 (13.5)	22 (18)	7 (7.5)	**0** **.** **019**
Pretreatment Total Cholesterol (mg/dL)	319 (290–356)	340 (316–378)	300 (275–311)	**<0** **.** **001**
Pretreatment LDL-C (mg/dL)	244 (210–277)	265 (246–293)	211 (200–229)	**<0** **.** **001**
Pretreatment Non-HDL-C (mg/dL)	263 (234–300)	282 (260–323)	239 (220–260)	**<0** **.** **001**
Pretreatment HDL-C (mg/dL)	52 (45–64)	54 (45–64)	50 (46–64)	0.509
Pretreatment Triglyceride (mg/dL)	171 (129–227)	178 (130–229)	170 (129–226)	0.821
Lp(a) (mg/dL)	16 (9–40)	18 (11–45)	14 (6–32)	**0** **.** **003**
Apo A-I (mg/dL)	162.9 ± 27.4	162.8 ± 28.9	163 ± 25.4	0.950
Apo B (mg/dL)	126.2 ± 32.1	130.7 ± 35.9	120.6 ± 25.5	**0** **.** **016**
Apo A-I/Apo B ratio	1.28 (1.1–1.7)	1.23 (1–1.7)	1.32 (1.1–1.7)	0.138
Lp(a) ≥ 30 mg/dL, *n* (%)	67 (31)	41 (34)	26 (28)	0.285
Lp(a) ≥ 50 mg/dL, *n* (%)	42 (19.5)	29 (24)	13 (14)	0.054
Apo B ≥ 130 mg/dL, *n* (%)	105 (49)	65 (54.2)	40 (42)	0.079
Aortic Valve Disease, *n* (%)	9 (4)	7 (6)	2 (2)	0.304
Aspirin Use, *n* (%)	37 (17)	27 (22.5)	10 (10.5)	**0** **.** **021**
Statin Use, *n* (%)	68 (32)	49 (41)	19 (20)	**0** **.** **001**
Ezetimibe Use, *n* (%)	7(3.5)	7 (6)	0	**0** **.** **018**
50% reduction in LDL-C level, *n* (%)	45 (21)	30 (25)	15 (16)	0.099
LDL-C < 70 mg/dL, *n* (%)	8 (4)	3 (2.5)	5 (5)	0.306

BMI: body mass index; CAD: coronary artery disease; Lp(a): lipoprotein (a); Apo: apolipoprotein; LDL-C: low-density lipoprotein-cholesterol; HDL-C: high-density lipoprotein-cholesterol; Stigmata: (tendon xanthoma, arcus cornealis, xanthelasma); * Obesity: BMI ≥ 30 kg/m^2^. Genetic test results were available for 46 patients (42 patients had LDL-R gene mutations and 4 patients had Apo B gene mutations).

**Table 2:** Distribution patterns of subclinical atherosclerosis and plaque characteristics.

Variables	Study Population (*n*: 215)	Definite Clinical Diagnosis (*n*: 120)	Probable Clinical Diagnosis (*n*: 95)	*p* Value
Individuals with Subclinical Atherosclerosis, *n* (%)	148 (69)	95 (79)	53 (56)	**<0** **.** **001**
Subclinical Atherosclerosis Distribution, *n* (%)	215	120	95	0.614
1 arterial bed	53 (25)	32 (27) a	21 (22) a	
2 arterial beds	58 (27)	40 (33) a	18 (19) a	
3 arterial beds	37 (17)	23 (19) a	14 (15) a	
Individuals with Carotid Plaque, *n* (%)	102 (47.5)	63 (52.5)	39 (41)	0.095
Maximum Carotid Plaque Thickness ≥ 1.5 mm, *n* (%)	102 54 (53)	63 32 (51)	39 22 (56.5)	0.581
Carotid Plaque Content *, *n* (%)	102	63	39	0.359
Type 1	19 (18)	9 (14) a	10 (26) a	
Type 2 and 3 (mix)	20 (20)	13 (21) a	7 (18) a	
Type 4	63 (62)	41 (65) a	22 (56) a	
Individuals with Femoral Plaque, *n* (%)	87 (40.5)	58 (48)	29 (31)	**0** **.** **008**
Maximum Femoral Plate Thickness ≥ 1.5 mm, *n* (%)	87 47 (54)	58 30 (52)	29 17 (59)	0.543
Femoral Plaque Content *, *n* (%)	87	58	29	0.06
Type 1	11 (13)	4 (7) a	7 (24) a	
Type 2 and 3 (mix)	14 (16)	11 (19) a	3 (10) a	
Type 4	62 (71)	43 (74) a	19 (66) a	
CAC Score ** ≥ 1, *n* (%)	188 90 (48)	103 59 (57)	85 31 (36.5)	**0.004**
CAC Score ** ≥ 100, *n* (%)	188 20 (11)	103 14 (13.5)	85 6 (7)	0.148

CAC: Coronary artery calcium. a: no significant difference was observed in subgroup analysis. *: Plaque characteristics were classified according to the Gray–Weale classification. **: CAC score was calculated over 188 patients.

**Table 3:** Variables demonstrating strong correlation with the extent of subclinical atherosclerosis.

	Study Population		Definite Clinical Diagnosis		Probable Clinical Diagnosis	
Variables	**r**	***p* Value**	**r**	***p* Value**	**r**	***p* Value**
Age (years)	0.431	**<0.001**	0.389	**<0.001**	0.404	**<0.001**
Pretreatment LDL-C (mg/dL)	0.328	**<0.001**	0.227	**0.013**	0.262	**0.01**
Pretreatment nonHDL-C (mg/dL)	0.368	**<0.001**	0.283	**0.002**	0.344	**0.001**
Apo A-I (mg/dL)	0.147	**0.031**	0.220	**0.016**	0.058	0.578
Apo B (mg/dL)	0.146	**0.033**	0.146	0.111	0.103	0.322
Lp(a) (mg/dL)	0.084	0.218	0.092	0.320	−0.037	0.724
DLCN Score	0.255	**<0.001**	0.115	0.211	0.175	0.090
Carotid Plaque Thickness (mm)	0.693	**<0.001**	0.653	**<0.001**	0.747	**<0.001**
Femoral Plaque Thickness (mm)	0.724	**<0.001**	0.690	**<0.001**	0.740	**<0.001**
CAC Score (HU)	0.800	**<0.001**	0.754	**<0.001**	0.819	**<0.001**

Apo: apolipoprotein; Lp(a): lipoprotein (a); CAC: coronary artery calcium; DLCN: Dutch Lipid Clinical Network; LDL-C: LDL: low-density lipoprotein-cholesterol; HDL: high-density lipoprotein-cholesterol; HU: Hounsfield Unit.

**Table 4:** ROC analysis of variables correlated with the extent of subclinical atherosclerosis.

Variables	AUC (%95 CI)	Cut-Off	Sensitivity	Specificity	*p* Value
Age (years)	0.732 (0.652–0.812)	≥55.5	65%	65%	**<0.001**
Femoral Plaque Thickness (mm)	0.899 (0.856–0.942)	≥0.75 mm	84%	84%	**<0.001**
CAC Score (HU)	0.891 (0.838–0.944)	≥19.5	81%	83%	**<0.001**
Carotid Plaque Thickness (mm)	0.872 (0.823–0.921)	≥1.15 mm	78.4%	79.3%	**<0.001**
Pretreatment LDL-C (mg/dL)	0.625 (0.529–0.726)	≥246.5	60%	58%	**0.018**
Pretreatmentnon HDL-C (mg/dL)	0.665 (0.574–0.757)	≥263.5	60%	61.3%	**0.02**
Lp(a) (mg/dL)	0.581 (0.478–0.684)	≥17.5	60%	58%	0.127

LDL: low-density lipoprotein-cholesterol; HDL: high-density lipoprotein-cholesterol; HU: Hounsfield Unit; Lp(a): lipoprotein (a); CAC: coronary artery calcium.

**Table 5:** Multivariable logistic regression analysis and independent predictors of subclinical atherosclerosis.

	Subclinical Atherosclerosis			Coronary Atherosclerosis			Carotid Artery Atherosclerosis			Femoral Artery Atherosclerosis		
Variables	Odds Ratio	%95 CI	*p* Value	Odds Ratio	%95 CI	*p* Value	Odds Ratio	%95 CI	*p* Value	Odds Ratio	%95 CI	*p* Value
Age (years)	1.134	1.078–1.191	**<0.001**	1.097	1.051–1.146	**<0.001**	1.092	1.051–1.135	**<0.001**	1.118	1.071–1.166	**<0.001**
Male Gender, *n* (%)	5.225	1.937–14.09	**0.001**	3.374	1.361–8.360	**0.009**	2.435	1.072–5.529	**0.033**	3.250	1.347–7.838	**0.009**
BMI (kg/m^2^)	0.925	0.848–1.009	0.078	0.986	0.908–1.070	0.730	0.939	0.867–1.016	0.118	0.959	0.883–1.042	0.325
Smoking, *n* (%)	1.055	0.512–2.174	0.885	1.495	0.748–2.989	0.255	1.426	0.746–2.726	**0.284**	2.521	1.261–5.040	**0.009**
Hypertension, *n* (%)	1.125	0.469–2.702	0.791	1.675	0.777–3.611	0.188	1.666	0.823–3.375	0.156	1.408	0.681–2.910	0.355
Diabetes, *n* (%)	4.079	1.006–16.55	**0.049**	1.021	0.386–2.698	0.967	3.084	1.171–8.127	**0.023**	1.285	0.513–3.218	0.593
Pretreatment LDL-C (mg/dL)	1.014	1.006–1.023	**0.001**	1.011	1.004–1.019	**0.004**	1.007	1.002–1.013	**0.012**	1.014	1.007–1.021	**<0.001**
Lp(a) > 30 mg/dL, *n* (%)	1.308	0.603–2.837	0.497	2.649	1.253–5.599	**0.011**	1.494	0.760–2.934	0.244	1.149	0.568–2.324	0.698
Apo A-l/Apo B ratio	0.442	0.203–0.965	**0.040**	0.636	0.311–1.303	0.216	0.420	0.210–0.842	**0.014**	0.736	0.371–1.463	0.382
	Hosmer-Lemeshow Test: 0.542 Nagelkerke R^2^ Value: 0.391			Hosmer-Lemeshow Test: 0.280 Nagelkerke R^2^ Value: 0.305			Hosmer-Lemeshow Test: 0.166 Nagelkerke R^2^ Value: 0.305			Hosmer-Lemeshow Test: 0.521 Nagelkerke R^2^ Value: 0.355		

BMI: Body Mass Index, LDL-C: Low Density Lipoprotein Cholesterol, Apo: Apolipoprotein, Lp(a): Lipoprotein (a), Cl: Confidence Interval.

## Data Availability

Data are available from the authors upon request.

## Abbreviations

The following abbreviations are used in this manuscript:

IMT	Intima-medial thickness
CAC	Coronary artery calcium
LAD	Left anterior descending coronary artery
Cx	Circumflex artery
RCA	Right coronary artery
Lp(a)	Lipoprotein a
Apo-A1	Apolipoprotein A1
Apo-B	Apolipoprotein B
PCSK-9	Proprotein convertase subtilisin/kexin type 9
BMI	Body mass index
CAD	Coronary artery disease
TG	Triglyceride
HU	Hounsfield Unit
LDL-C	Low-density lipoprotein-cholesterol
HDL-C	High-density lipoprotein-cholesterol
FH	Familial hypercholesterolemia
DLCN	Dutch Lipid Clinical Network
ASCVD	Atherosclerotic Cardiovascular Disease
ACS	Acute coronary syndrome
CABG	Coronary artery bypass grafting
USG	Ultrasonography
